# Biocompatibility and odontogenic potential of an indigenous apitherapeutic pulp capping agent: A comparative in vitro analysis using hDPSCs

**DOI:** 10.1016/j.jobcr.2025.11.008

**Published:** 2025-11-25

**Authors:** Ketaki Turbatmath, S. Delphine Priscilla Antony, Raghunandhakumar Subramanian

**Affiliations:** a Department of Conservative Dentistry and Endodontics, Saveetha Dental College and Hospitals, Saveetha Institute of Medical and Technical Sciences (SIMATS), Saveetha University, Chennai, 600077, Tamil Nadu, India; b Cancer and Stem Cell Research Laboratory, Department of Pharmacology, Saveetha Dental College and Hospitals, Saveetha Institute of Medical and Technical Sciences (SIMATS), Saveetha University, Chennai, 600077, Tamil Nadu, India

**Keywords:** Apitherapeutic pulp guard, Cytocompatibility, hDPSCs, MTT assay, Odontogenic differentiation, qPCR

## Abstract

**Introduction:**

This study aimed to evaluate the cytocompatibility and odonto-inductive potential of an indigenously developed apitherapeutic pulp capping agent Api-Therapeutic Pulp Guard (ATPG) by assessing cell viability and dentinogenic gene expression using human dental pulp stem cells (hDPSCs)

**Materials and methods:**

hDPSCs were exposed to eluates of ATPG and compared with calcium hydroxide (RC-Cal) and control. MTT assay was used to assess cell viability, LIVE/DEAD staining was performed using calcein-AM and ethidium homodimer, and quantitative real-time PCR (qPCR) evaluated DSPP gene expression. This comprehensive approach provided complementary insights into cell metabolic activity, membrane integrity, and odontogenic potential of the test materials.

**Results:**

ATPG exhibited high cell viability (96.52 %), significantly greater than calcium hydroxide (84.25 %) (*p* < 0.01). LIVE/DEAD assay confirmed better membrane integrity with a predominance of green (live) stained cells. qPCR showed a 3.6-fold upregulation of DSPP gene expression in ATPG-treated cells, suggesting strong odontogenic differentiation.

**Conclusion:**

The ATPG formulation demonstrated superior biocompatibility and odontogenic potential compared to conventional calcium hydroxide, underscoring its promise for translation in regenerative endodontic therapy.

## 1 Introduction

Maintaining the vitality of the dental pulp through minimally invasive therapeutic strategies has become a central goal in conservative dentistry.^1^ Vital pulp therapy (VPT) is a conservative clinical approach aimed at preserving the health and function of the dental pulp following mechanical, traumatic, or carious exposure.^2^ Direct pulp capping, a key VPT procedure, involves placing a bioactive material directly over exposed pulp tissue to stimulate the formation of a dentin bridge, thereby protecting pulp vitality and promoting healing by inducing reparative or tertiary dentin.^3^^,^^4^ This process is largely dependent on the migration and differentiation of human dental pulp stem cells (hDPSCs) into odontoblastic-like cells, which are crucial for reparative dentin formation.^5^ The success of such interventions largely hinges on the material's ability to be non-cytotoxic and to promote regeneration through dentin bridge formation, which is critically governed by the activity of human dental pulp stem cells (hDPSCs).^6^^,^^7^

Historically, calcium hydroxide (Ca(OH)2) has been considered the “gold standard” for direct pulp capping due to its high pH, antimicrobial properties, and ability to stimulate reparative dentin formation.^8^ However, Ca(OH)2 exhibits significant limitations, including high solubility, weak mechanical properties, poor sealing ability, potential for long-term failure, stimulation of coagulative necrosis, and initial cytotoxicity, often leading to the formation of porous dentin bridges with tunnel defects susceptible to bacterial invasion.^9^^,^^10^ To address these shortcomings, Mineral Trioxide Aggregate (MTA) emerged as a superior alternative, offering improved sealing ability, high biocompatibility, and bioinductive properties, inducing a more homogeneous, localized, and thicker dentin bridge compared to Ca(OH)2.^11^ Despite its advantages, MTA also has drawbacks, such as a prolonged setting time, difficult handling properties, high cost, and the potential for tooth discoloration, particularly with gray MTA formulations.^12^ Newer calcium silicate-based materials like Biodentine and TheraCal LC have also been introduced, aiming to overcome some of MTA's limitations by offering faster setting times and improved handling.^13^^,^^14^ However, some resin-modified materials like TheraCal LC have been reported to show cytotoxicity due to residual monomers. Despite these advancements, limitations related to cost, handling, setting time, and aesthetic properties persist, necessitating the exploration of novel, biologically enriched alternatives.^15^

The search for an ideal pulp capping material continues, emphasizing the need for agents that not only prevent bacterial infiltration and promote dentin bridge formation but also trigger minimal inflammation and enhance hDPSC differentiation effectively. This has led to a growing interest in natural biomaterials with intrinsic biological properties.^16^ Apiarian products, such as propolis and royal jelly, have a long history of medicinal use due to their antimicrobial, anti-inflammatory, antioxidant, and tissue-regenerating properties.^17^ Propolis is rich in flavonoids and phenolic compounds, known for their antimicrobial and antioxidant activities, and capacity to enhance wound healing and tissue regeneration.^18^ Royal jelly, on the other hand, contains bioactive peptides, lipids, and vitamins that promote tissue repair, reduce inflammation, and accelerate recovery.^19^^,^^20^ When formulated into pulp capping agents, apitherapeutic compounds such as propolis and royal jelly may offer an ideal balance between biocompatibility, angiogenic modulation, and dentinogenic stimulation, supported by their antioxidant, anti-inflammatory, and growth-factor modulating properties.^21^^,^^22^ This biological synergy positions apitherapeutic formulations as promising candidates for regenerative endodontic applications, bridging the gap between natural biomaterials and contemporary calcium silicate-based cements. Dental pulp stem cells, owing to their high proliferative and differentiation capacity, serve as a relevant cellular model to assess the biological performance of such biomaterials. Despite their demonstrated potential, there is a paucity of studies rigorously comparing the performance of apiarian products to conventional materials like Ca(OH)2 and MTA in dental applications.

In vitro models play an essential role in evaluating the cytotoxicity and regenerative potential of dental biomaterials prior to in vivo translation.^23^ Among the various assays, the MTT assay is a well-established method for determining mitochondrial activity and cell viability, while LIVE/DEAD staining enables visualization of viable versus necrotic/apoptotic cells based on membrane integrity.^24^ In parallel, quantitative reverse transcription-polymerase chain reaction (qRT-PCR) of dentinogenesis-related markers such as dentin sialophosphoprotein (DSPP) provides insight into the odontogenic potential of pulp capping materials.^25^

This study helps to fill this gap by focusing on the *in vitro* biocompatibility and odontogenic potential of an indigenously developed apiarian-based pulp capping agent, named Api-therapeutic Pulp Guard, in comparison to conventional materials using human dental pulp stem cells (hDPSCs). The comprehensive *in vitro* evaluation is crucial for understanding its cellular interactions and bioinductive capabilities before further in vivo and clinical assessments. The study aims to comprehensively evaluate the cytocompatibility and odontogenic differentiation capacity of a novel api-therapeutic pulp capping formulation containing royal jelly and propolis in comparison with a commercially available calcium hydroxide-based material (Dentsply Dycal). The assessment was performed using MTT assay, LIVE/DEAD cell viability assay, and qRT-PCR for DSPP gene expression, using hDPSCs as the cellular model. The outcomes of this study provide a foundational framework for validating the biological efficacy of apitherapeutic agents in vital pulp therapy.

## 2 Materials and methods

### 2.1 Pulp capping agent preparation

A novel apitherapeutic pulp capping formulation, Api-Therapeutic Pulp Guard (ATPG), was developed using a synergistic combination of royal jelly and propolis with two naturally derived bioactive substances known for their regenerative, anti-inflammatory, and antimicrobial properties.^26^

Lyophilized propolis powder was first dissolved in 95 % ethanol (analytical grade) to form a concentrated ethanolic extract. Separately, lyophilized royal jelly powder was reconstituted in sterile distilled water. The two liquids were combined in a 1:1 ratio to obtain a homogenous bioactive base. To transform this liquid into a semi-solid matrix, agar-agar powder (2 % w/v) was incrementally added under controlled stirring at 60 °C to ensure uniform dispersion.

The final paste was homogenized until a stable, consistent consistency was achieved. The pH was adjusted to 6.8–7.2 using 0.1 N NaOH or HCl to simulate physiologic conditions optimal for pulp tissue compatibility. The ATPG formulation was sterilized by gamma irradiation and stored in sterile, airtight containers at 4 °C until further use.

### 2.2 Human dental pulp stem cell (hDPSC) culture

Human dental pulp stem cells (hDPSCs) were isolated from the dental pulps of freshly extracted, non-carious human third molars obtained with informed consent under approved institutional ethical guidelines. All procedures were carried out at the Cancer and Stem cell Research Lab, Department of Pharmacology, Saveetha Dental College, Chennai, under sterile laminar airflow conditions to maintain asepsis throughout the process. The extracted teeth were immediately placed in sterile Hank's Balanced Salt Solution (HBSS) supplemented with 1 % antibiotic–antimycotic solution and transported to the laboratory within 30 min of extraction. Pulp tissues were retrieved using a sterile barbed broach under aseptic conditions and subsequently subjected to enzymatic digestion with collagenase type I and dispase for cell isolation.

The cells were cultured in Dulbecco's Modified Eagle Medium (DMEM, Gibco, USA) supplemented with 10 % fetal bovine serum (FBS, Invitrogen, USA), 1 % penicillin-streptomycin, and 1 % L-glutamine, and incubated at 37 °C in 5 % CO_2_ and 95 % humidity. Subculturing was performed upon reaching 80–90 % confluency, and cells from passage 4 were selected for all experiments.

Flow cytometric analysis was performed to confirm the mesenchymal phenotype of hDPSCs, showing positive expression of CD73, CD90, CD105, and CD44, and negative expression for hematopoietic markers CD34, CD45, and CD19. Additionally, cells exhibited multipotent differentiation capacity into osteogenic, adipogenic, and chondrogenic lineages, further confirming stem cell characteristics.

### 2.3 Cytocompatibility assessment – MTT assay

Cell viability was assessed using the MTT [3-(4,5-dimethylthiazol-2-yl)-2,5-diphenyltetrazolium bromide] assay, which measures mitochondrial metabolic activity as an indicator of cytocompatibility following exposure to material eluates. hDPSCs were seeded into 96-well plates pre-coated with poly-L-lysine at a density of 5 × 10^3^ cells/well, which ensured optimal metabolic activity without over-confluence. Material extracts (eluates) of Api-Therapeutic Pulp Guard, Dycal (calcium hydroxide), and a negative control (untreated) were prepared by incubating 15 mg of each material in 1 mL of culture medium for 24 h at 37 °C.

Subsequently, 10 μL of MTT solution (10 mg/mL stock) was added to each well to achieve a final concentration of 1 mg/mL in 100 μL total volume and incubated for 4 h at 37 °C in 5 % CO_2_. Formazan crystals were dissolved in 100 μL dimethyl sulfoxide (DMSO), and the plate was gently shaken for 5 min before measuring absorbance at 570 nm with a reference wavelength of 630 nm using a Synergy Hybrid Multi-Mode Reader (BioTek, USA). Cell viability (%) was calculated relative to the control group. Positive (0.1 % Triton X-100-treated cells) and blank wells (medium + MTT without cells) were included to determine the assay's dynamic range and correct background absorbance. All measurements were performed in triplicate.

$$cell\;viability\;\left(\%\right)=\frac{OD\left(test\;sample\right)-OD\left(blank\right)}{OD\left(PC\right)-OD\left(blank\right)}\times 100$$

### 2.4 Live/dead cell viability assay

To qualitatively assess cytocompatibility and membrane integrity, a LIVE/DEAD viability assay was conducted using Calcein-AM (2 μM) and Ethidium homodimer-1 (EthD-1, 4 μM) (Invitrogen™ LIVE/DEAD™ Cell Imaging Kit). For each group, hDPSCs were seeded at a density of 1 × 10^5^ cells/well in 2 mL of complete medium and allowed to adhere overnight. To validate assay responsiveness, a positive control (0.1 % Triton X-100–treated cells) was used to represent membrane-compromised cells, and a negative control (untreated cells) represented fully viable cells.

After 24 h exposure to material eluates, cells were stained with 2 μM calcein-AM and 4 μM ethidium homodimer-1 for 30 min at 37 °C in the dark. Following staining, coverslips were rinsed twice with sterile PBS to remove excess dye and mounted in DAPI-containing antifade medium for nuclear counterstaining.

Images were captured using a Zeiss Axio Observer fluorescence microscope at 20 × magnification equipped with FITC and TRITC filter sets. Identical exposure times and gain settings were maintained across all samples to ensure consistency. Green fluorescence indicated live cells with intact membranes, while red fluorescence represented dead cells with compromised membranes. Three representative fields per sample were imaged and analyzed.

### 2.5 Odontogenic differentiation – DSPP gene expression (qRT-PCR)

To assess the odontogenic potential of the test materials, the expression of dentin sialophosphoprotein (DSPP) was measured using quantitative real-time PCR (qRT-PCR). After 24-h treatment, total RNA was extracted using TRIzol reagent (Thermo Fisher Scientific, USA). RNA purity and concentration were assessed using a NanoDrop 2000 spectrophotometer. cDNA synthesis was performed from 500 ng RNA using the PrimeScript RT Reagent Kit (Takara, Japan). qRT-PCR was conducted using KAPA SYBR Fast Master Mix (Sigma-Aldrich) in a Bio-Rad CFX Connect Real-Time PCR system. The following primers were used.

The thermal cycling program included an initial denaturation at 95 °C for 3 min, followed by 40 cycles of 95 °C for 3 s and 58 °C for 30 s. Gene expression was normalized to GAPDH and analyzed using the 2^−ΔΔCt method. Experiments were conducted in duplicate for each condition.

### 2.6 Statistical analysis

All experiments were conducted in triplicate (n = 3 independent biological replicates), each comprising three technical repeats to ensure data reliability. Results were expressed as mean ± standard deviation (SD). Statistical analyses were performed using one-way ANOVA followed by Tukey's post hoc test to compare intergroup differences. Data normality and variance homogeneity were confirmed prior to analysis. All statistical procedures were executed using GraphPad Prism v 8.0 (GraphPad Software, CA, USA), with a p-value <0.05 considered statistically significant.

## 3 Results

### 3.1 Cytocompatibility evaluation – MTT assay

The cytocompatibility of the tested pulp capping agents was quantitatively assessed using the MTT assay, which measures mitochondrial dehydrogenase activity as an indicator of cell viability.

Human dental pulp stem cells (hDPSCs) treated with eluates from Api-Therapeutic Pulp Guard showed a high level of viability (96.67 % ± 0.08), closely approximating the control group (100 % ± 0.06). In contrast, cells exposed to Dycal exhibited reduced viability (84.50 % ± 0.09), indicating a comparatively higher cytotoxic effect.

Statistical analysis via one-way ANOVA followed by Tukey's post hoc test revealed that the difference in cell viability between Dycal and the control was statistically significant (p < 0.01), while the ATPG group did not significantly differ from the control group (p > 0.05), suggesting excellent cytocompatibility of the apitherapeutic formulation (see Table 1).

**Table 1:** The Primers used for RT-PCR analysis.

Gene	Forward Primer (5′–3′)	Reverse Primer (5′–3′)
DSPP	CACAAGGGAGAAGGGAATG	TGCCATTTGCTGTGATGTTT
GAPDH	GAGTCAACGGATTTGGTCGT	TTGATTTTGGAGGGATCTCG

DSPP: Dentin Sialophosphoprotein; GAPDH: Glyceraldehyde 3-phosphate dehydrogenase.

Primer sequences were used to amplify target genes for odontogenic differentiation. GAPDH was employed as the housekeeping gene for normalization. DSP primers were designed to specifically amplify odontogenic gene expression. All primers were validated for specificity and efficiency prior to the amplification process.

**Fig. 1:**
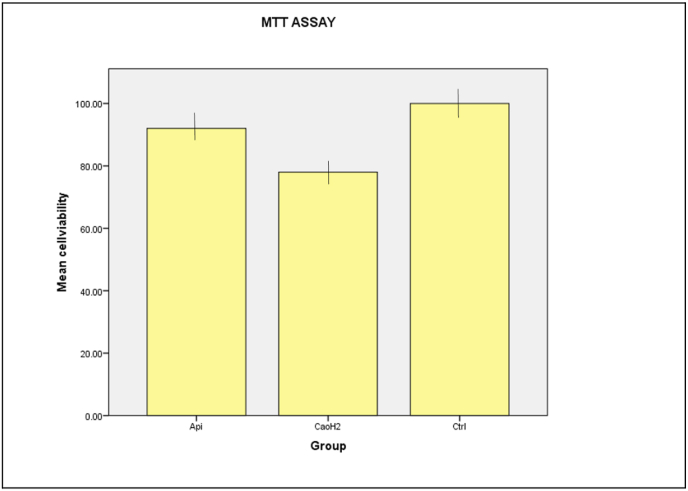
Bar Graph Illustrating Percentage Cell Viability by MTT Assay for All Experimental Groups. Higher viability in the control and api-therapeutic group (Api) compared to Dycal (calcium hydroxide (CaOH2)) indicates better cytocompatibility of the experimental formulation. Error bars represent standard deviation.

**Fig. 2:**
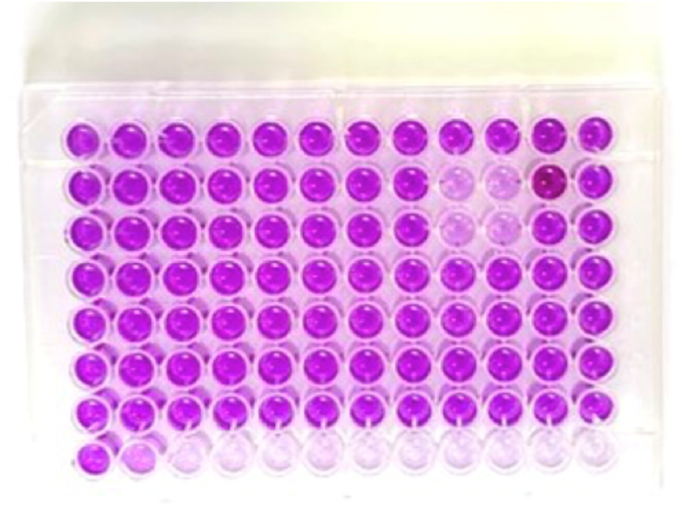
Representative 96-well microplate from MTT assay showing purple formazan formation, indicative of viable cells. (For interpretation of the references to color in this figure legend, the reader is referred to the Web version of this article.)

As depicted in Fig. 1, the bar graph illustrates the relative percentage of viable cells across all groups, with ATPG maintaining cell viability nearly equivalent to the untreated control. Fig. 2 shows representative images of the MTT assay procedure and formazan formation, visually confirming the metabolic activity retained by cells in the ATPG group. The detailed absorbance values, calculated percentage viability, and standard deviations for all three groups are presented in Table 2.

**Table 2:** MTT assay showing cytocompatibility of api-therapeutic pulp guard compared to control and calcium hydroxide.

	MTT-		ATPG
	Control	Dycal	
O.D	0.818	0.723	0.811
O.D	0.741	0.628	0.714
O.D	0.692	0.551	0.651
%	100	88.38631	99.14425
	100	84.75034	96.35628
	100	79.62428	94.07514
Mean	100	84.25364	96.52522
SD	0	3.594285	2.072901
p-Value	0	0.006489	0.001142

OD: Optical Density; SD: Standard Deviation; ATPG: Api-Therapeutic Pulp Guard.

Cell viability (%) was calculated relative to the control group (100 %). Measurements were taken after 24-h incubation with material eluates. Statistical analysis was performed using one-way ANOVA with Tukey's post hoc test; p < 0.05 was considered significant.

These results demonstrate that the novel ATPG is non-cytotoxic and supports cell viability, making it a promising candidate for direct pulp capping applications.

### 3.2 LIVE/DEAD cell viability assay

**Fig. 3:**
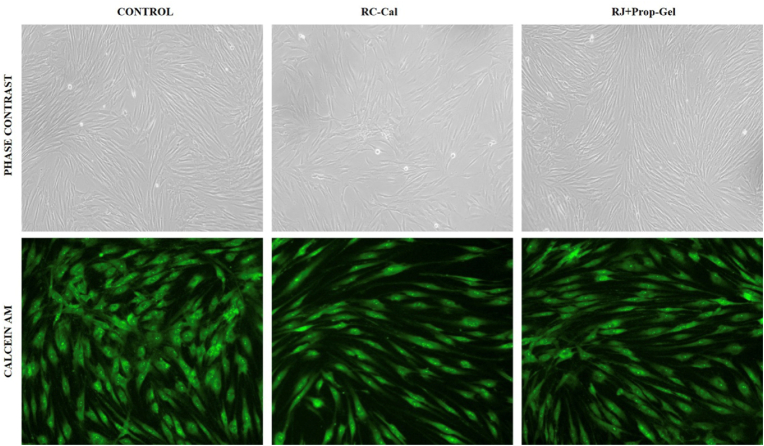
Live/Dead cell assay using Calcein-AM staining observed under phase contrast (top row) and fluorescent microscopy (bottom row) at 20× magnification. These observations validated the biocompatibility of ATPG and its favorable interaction with pulp stem cells, with minimal evidence of membrane damage or cell death.

Qualitative assessment using Calcein-AM and Ethidium Homodimer-1 staining further confirmed the results obtained from the MTT assay. As shown in Fig. 3, fluorescence microscopy images demonstrate the relative proportion of live (green) and dead (red) cells across different experimental groups: a)Control group: Predominantly green fluorescent cells, indicating healthy and viable hDPSCs. b)ATPG: A high proportion of green-stained cells with intact morphology, comparable to the control group. c)Dycal group: A noticeable increase in red-stained (dead) cells, indicating compromised membrane integrity and reduced cytocompatibility.

### 3.3 Odontogenic differentiation – DSPP gene expression (qRT-PCR)

**Fig. 4:**
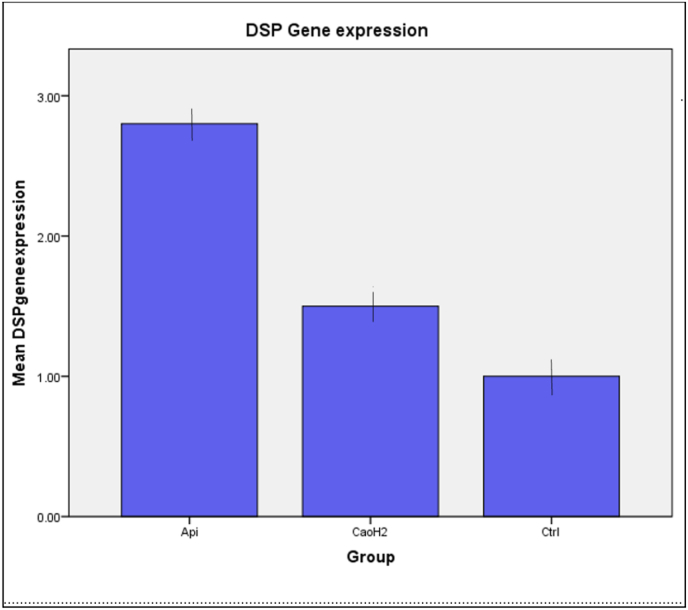
Bar Graph Illustrating the Relative Expression Levels of DSP Gene Among Experimental Groups. Highest in Api-therapeutic group (API) followed by calcium hydroxide group CaOH2.

To assess the odontogenic potential of the materials, qRT-PCR was conducted to measure the expression levels of dentin sialophosphoprotein (DSPP), a key marker for odontoblast-like differentiation. As depicted in Fig. 4, the bar graph illustrates the relative fold change in DSPP expression among the three groups: a)Control group: Baseline DSPP expression was normalized to 1.0. b)Dycal group: Showed a moderate increase in DSPP expression (∼1.8–2.5-fold). c)ATPG: Exhibited robust upregulation of DSPP gene expression (∼3.6-fold) compared to the control, indicating a strong stimulatory effect on odontogenic differentiation.

**Fig. 5:**
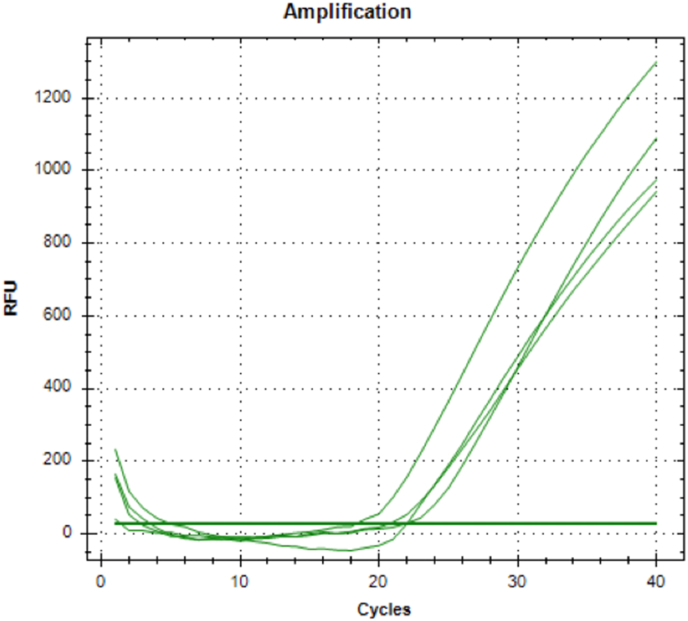
Amplification Plot for DSP Gene Expression Analysis. The exponential increase in fluorescence indicates successful amplification. Lower cycle threshold (Ct) values correlate with higher initial gene expression levels. The curve confirms the presence and relative quantification of dentin sialoprotein (DSP) expression in treated samples.

Further visual confirmation of the qRT-PCR assay and amplification specificity is presented in Fig. 5, which displays representative melt curves and amplification plots confirming the reliability of the data. The quantitative values, mean fold changes, and standard deviations are summarized in Table 3, supporting the statistical relevance of the observed differences. This significant increase in gene expression levels in the ATPG group highlights its bioinductive potential and capacity to promote odontoblast-like activity in hDPSCs. These findings suggest that ATPG not only preserves cell viability but also enhances reparative dentinogenesis at the molecular level.

**Table 3:** Relative expression levels of DSP gene.

	Control		Dycal		ATPG			
DSP	1	1	1.4	1.6	2.5	3.1	3.4	3.6

DSP: Dentin Sialophosphoprotein; ATPG: Api-Therapeutic Pulp Guard.

Fold changes in gene expression were calculated using the 2^−ΔΔCt method, normalized to GAPDH.Experiments were performed in duplicate for each condition.

## 4 Discussion

Vital pulp therapy (VPT) represents a biologically driven approach to preserve the vitality of the pulp-dentin complex following reversible injury and promote reparative dentinogenesis.^2^ The long-term success of VPT is intrinsically linked to the bioactivity and biocompatibility of the chosen pulp capping agent. While traditional materials such as calcium hydroxide (CH) and mineral trioxide aggregate (MTA) have been widely utilized, their limitations in biological signaling, tissue compatibility, solubility, mechanical properties, inflammatory response, cost and regenerative potential have prompted the exploration of novel alternatives.^11^

Calcium hydroxide, though effective in stimulating initial reparative dentin formation, exhibits high alkalinity (pH > 12) that can lead to superficial necrosis of the pulp tissue, cytotoxic effects on neighboring cells, and inconsistent dentin bridge formation.^24^ MTA, while superior in terms of sealing ability and bioactivity, is constrained by tooth discoloration, high cost, prolonged setting time, and difficulty in handling, particularly in pediatric or resource-limited settings.^11^

This study presents a comprehensive *in vitro* analysis of an indigenously developed Api-therapeutic Pulp Guard, demonstrating that ATPG exhibited superior cytocompatibility and odontogenic potential when compared with calcium hydroxide. The results of the study align with the hypothesis that apitherapeutic formulations can support cellular vitality through antioxidant and bio-modulatory mechanisms.

When benchmarked against contemporary bioceramic-based materials such as Mineral Trioxide Aggregate (MTA) and Biodentine, the Api-Therapeutic Pulp Guard (ATPG) demonstrates comparable or enhanced cytocompatibility and odontogenic gene upregulation. Unlike calcium silicate matrices that primarily induce bioactivity through ion release, ATPG incorporates naturally derived bioactive compounds with intrinsic antioxidant, anti-inflammatory, and angiogenic properties, offering a dual mechanism of pulp regeneration.

Recent studies have underscored the role of vascular endothelial growth factor (VEGF) and fibroblast growth factor-2 (FGF2) in early pulp healing and dentin bridge formation; the apitherapeutic components of ATPG may stimulate these pathways through modulation of oxidative stress and cytokine signaling.

From a translational standpoint, these findings highlight ATPG as a biologically driven alternative capable of promoting cellular vitality, dentinogenesis, and vascular support key determinants for successful vital pulp therapy and regenerative endodontic outcomes.

The Api-therapeutic Pulp Guard leverages the inherent therapeutic properties of royal jelly and propolis. Royal jelly, rich in major royal jelly proteins (MRJPs) and bioactive peptides like royalactin, is known to enhance cellular proliferation, modulate immune responses, and stimulate odontoblastic differentiation, potentially through TGF-β and Wnt/β-catenin signaling pathways.^17^ Propolis, abundant in flavonoids and phenolic acids, contributes potent antimicrobial, anti-inflammatory, antioxidant, and wound-healing properties, supporting tissue regeneration and angiogenesis via VEGF upregulation.^27^ These bee-derived biomolecules possess well-documented antioxidant, anti-inflammatory, and regenerative properties, attributed to their polyphenolic content, flavonoids, fatty acids, MRJPs (Major Royal Jelly Proteins), and CAPE (Caffeic Acid Phenethyl Ester).^28^

The physical and chemical characterization of the Api-therapeutic Pulp Guard through SEM and FTIR confirmed a porous, crystalline-fibrous matrix and the retention of these bioactive functional groups, which are favorable for cell adhesion, controlled release of active compounds, and beneficial material-cell interactions. The agar-agar base further offers physiological pH (6.8–7.2), favorable viscosity, and stable scaffold properties.

### 4.1 In vitro biocompatibility via MTT assay

The MTT assay, a gold standard for cytotoxicity evaluation, relies on mitochondrial succinate dehydrogenase-mediated reduction of tetrazolium salts to formazan.^7^ In this study, hDPSCs exposed to ATPG eluates demonstrated 96.52 % ± 0.08 viability, statistically indistinguishable from control (100 %) and significantly greater than Dycal (84.25 % ± 0.09, *p* < 0.01). This suggests that ATPG maintains mitochondrial integrity and metabolic activity, reinforcing its non-cytotoxic profile. Previous studies have shown that flavonoids such as chrysin and pinocembrin in propolis protect against oxidative mitochondrial stress, which could explain the observed metabolic preservation.^18^

This finding is crucial as viable hDPSCs are essential for the reparative processes of the pulp. In contrast, studies on other materials show varying cytotoxic effects; for instance, Dycal has been consistently reported exhibiting lower cell viability and higher cytotoxicity compared to other contemporary pulp capping agents like MTA and Biodentine.

### 4.2 Membrane integrity via LIVE/DEAD cell assay

LIVE/DEAD imaging using Calcein-AM and Ethidium Homodimer-1 provided visual confirmation of cell viability and membrane integrity. hDPSCs treated with ATPG showed a predominance of Calcein-positive (green) cells, suggesting intact membranes and active esterase function. In contrast, Dycal-treated cells exhibited increased EthD-1 uptake (red fluorescence), indicating membrane disruption and apoptotic or necrotic signaling. This corroborates the hypothesis that the neutral pH and antioxidant buffering capacity of ATPG mitigate membrane destabilization. Similar membrane-stabilizing effects of propolis-derived phenolics have been demonstrated in gingival fibroblast cultures.^29^

The odontogenic potential of a pulp capping agent refers to its ability to stimulate hDPSCs to differentiate into odontoblast-like cells, leading to reparative dentin formation. The significant 3.6-fold upregulation of dentin sialophosphoprotein (DSP) gene expression by the Api-therapeutic Pulp Guard is a strong indicator of its bioinductive properties at the molecular level. DSP is a well-established marker for odontoblastic differentiation. This suggests that the Api-therapeutic Pulp Guard not only supports cell survival but actively promotes the differentiation necessary for dentin bridge formation. This aligns with the known mechanisms of royal jelly and propolis in stimulating odontoblastic differentiation and enhancing mineralization. While some conventional materials like MTA and Biodentine also demonstrate odontogenic potential, and materials like Activa Bioactive upregulate DSPP, the specific magnitude of DSP upregulation by the Api-therapeutic Pulp Guard highlights its promise. Some studies indicate that while some materials like Dycal may increase ALP activity (an early mineralization marker), they might not consistently promote overall odontogenic differentiation or lead to optimal mineralization.

### 4.3 Odontogenic differentiation via DSPP gene expression

The expression of Dentin Sialophosphoprotein (DSPP), a late odontoblastic marker critical for dentinogenesis and matrix mineralization, was evaluated using qRT-PCR. hDPSCs treated with ATPG demonstrated a 3.6-fold upregulation of DSPP, significantly greater than RC-Cal (∼2.15-fold) and control. This suggests that ATPG promotes lineage-specific differentiation of dental pulp progenitors toward an odontoblast-like phenotype.

Royal jelly is known to upregulate TGF-β1 and Wnt/β-catenin signaling pathways, both essential in odontoblastic differentiation. Royalactin, a unique peptide in royal jelly, activates EGFR and downstream MAPK cascades, further stimulating proliferation and differentiation.^19^ Similarly, CAPE from propolis acts as a pro-odontogenic bioactive, modulating transcription factors such as RUNX2 and Dlx5.^4^

### 4.4 Zebrafish biosafety validation

These *in vitro* results are further substantiated by our previously published zebrafish embryo toxicity study, which confirmed that ATPG exhibited no teratogenicity or systemic toxicity. The survival, hatching, and heart rate parameters in zebrafish embryos treated with ATPG were comparable to controls and significantly better than those treated with calcium hydroxide. Given the zebrafish model's established translational relevance in toxicogenomic screening, this reinforces ATPG's biosafety for potential clinical use.

Furthermore, antimicrobial and antibiofilm efficacy are crucial for preventing secondary infection and ensuring the long-term success of vital pulp therapy. The Api-therapeutic Pulp Guard's demonstrated antibacterial activity against *E. faecalis* and *S. aureus*, along with its potent antibiofilm effects, including significant reductions in CFU counts, biofilm biomass, and metabolic activity, are highly relevant for combating common endodontic pathogens. The phenolic components of propolis are thought to contribute to these effects by interfering with bacterial membrane integrity and metabolism within biofilms.

The present investigation integrates multiple complementary endpoints, cell viability (MTT), membrane integrity (LIVE/DEAD), and odontogenic gene expression (DSPP), providing a holistic evaluation of biocompatibility and bioinductive potential. The use of standardized protocols, triplicate biological and technical repeats, and consistent imaging conditions enhances the reproducibility and reliability of the results, underscoring the strength of the current experimental design.

### 4.5 Limitations

Despite promising findings, this study has several limitations: Only one odontogenic gene marker (DSPP) was evaluated; future work should incorporate a broader panel (DMP-1, OCN, ALP, RUNX2, BSP) to confirm multi-lineage differentiation. Limitations specific to the *in vitro* nature of this study must be acknowledged. While *in vitro* models provide controlled environments for initial biocompatibility and bioactivity assessments, they cannot fully replicate the complex physiological conditions of the oral cavity and pulp tissue, including intricate cellular interactions, blood supply, immune responses, and mechanical stresses. The variability inherent in donor-derived hDPSCs could also influence reproducibility.

Longitudinal studies tracking mineral nodule formation, using Alizarin Red or Von Kossa staining, were not performed. Although zebrafish models provide rapid biosafety assessment, extrapolation to human in vivo contexts may require pulp organ culture models or animal studies with histomorphometry. Mechanical strength, compressive resistance, and setting time were not evaluated; these are critical for functional durability in clinical conditions.

### 4.6 Future scope

Advanced molecular assays such as RNA-seq or proteomic profiling can elucidate signaling networks activated by ATPG. Incorporation of 3D organoid or tooth slice models will allow for better simulation of the pulp-dentin microenvironment. Comparative trials against newer silicate-based materials (e.g., Biodentine, Theracal) will position ATPG within the current material landscape. Exploration of ATPG as a carrier for stem cells or growth factors (e.g., BMP-2, PDGF) may further enhance its regenerative potential. Future research should aim to address these limitations through further in vivo animal studies with histomorphometric and micro-CT evaluations, and eventually, well-designed clinical trials. In vivo histological validation using rodent or large animal pulp exposure models is essential to confirm dentin bridge formation. Future studies should also explore the synergistic incorporation of apitherapeutic components within calcium-silicate-based scaffolds to combine the bioactivity of silicate cements with the antioxidant and angiogenic potential of bee derived compounds. Such hybrid formulations could enhance dentin bridge architecture, vascular integration, and pulp vitality.

Long-term in-vivo and clinical studies are warranted to evaluate outcomes such as pulp sensibility preservation, dentin bridge thickness, and periapical healing, which would further establish the translational relevance of the Api-Therapeutic Pulp Guard.

Overall, the findings of this study support the potential of Api-Therapeutic Pulp Guard as a reproducible, biologically active material capable of complementing current regenerative strategies.

## 5 Conclusion

This study comprehensively evaluated the cytocompatibility and odontogenic potential of an indigenously developed Api-Therapeutic Pulp Guard (ATPG), a novel pulp capping agent derived from royal jelly and propolis, using a series of *in vitro* and biosafety assays. The formulation demonstrated excellent biocompatibility with high cell viability (96.67 %) in the MTT assay, preservation of cell membrane integrity via LIVE/DEAD staining, and a significant 3.6-fold upregulation of DSPP gene expression, confirming its potential to induce odontoblastic differentiation in hDPSCs.

The material's favorable cytocompatibility can be attributed to the antioxidant, anti-inflammatory, and regenerative properties of its apitherapeutic components, further supported by its neutral physiological pH and stable agar-based delivery system. In vivo validation using the zebrafish embryo toxicity model revealed no teratogenicity or systemic toxicity, reinforcing its biosafety profile. Compared to conventional pulp capping agents such as calcium hydroxide, which exhibited significantly lower cell viability and increased cytotoxicity, ATPG emerges as a biologically favorable, non-toxic alternative that supports both pulp cell survival and odontogenic differentiation hallmarks of successful vital pulp therapy.

While the present findings are promising, further studies are needed to validate its mechanical properties, mineralization potential, and long-term performance under clinical conditions. Nonetheless, ATPG presents as a cost-effective, naturally derived, and regenerative material with strong potential for translation into minimally invasive pulp therapy protocols.

## Patient's/Guardian's consent

Not applicable.

## Patient consent statement

This study did not involve any human participants, patients, or legal guardians. Therefore, no informed consent was required or obtained.

## Ethical clearance

SRB/SDC/ENDO-2206/24/474.

## Funding statement

This research did not receive any specific grant from funding agencies in the public, commercial, or not-for-profit sectors.

## Declaration of competing interest

The authors declare that they have no known competing financial interests or personal relationships that could have appeared to influence the work reported in this paper.
